# Distinguishing central precocious puberty from nonprogressive breast development: adiposity and DXA body composition

**DOI:** 10.1210/jendso/bvag134

**Published:** 2026-06-22

**Authors:** Daniela Fava, Amanda Casirati, Maria Grazia Calevo, Alessia Angelelli, Alessia Pepe, Nadia Gabriella Maiorano, Lucia Acquarone, Chiara Santucci, Claudia Caridi, Valentina Tosto, Marta Panciroli, Caterina Tedesco, Flavia Napoli, Anna Elsa Maria Allegri, Giuseppa Patti, Alessandro Naim, Roberto Gastaldi, Mohamad Maghnie, Natascia Di Iorgi

**Affiliations:** Pediatric Endocrinology Unit, Department of Pediatrics, IRCCS Istituto Giannina Gaslini, Genoa 16147, Italy; Pediatric Endocrinology Unit, Department of Pediatrics, IRCCS Istituto Giannina Gaslini, Genoa 16147, Italy; Epidemiology and Biostatistics Unit, Scientific Directorate, IRCCS Istituto Giannina Gaslini, Genoa 16147, Italy; Pediatric Endocrinology Unit, Department of Pediatrics, IRCCS Istituto Giannina Gaslini, Genoa 16147, Italy; Department of Neuroscience, Rehabilitation, Ophthalmology, Genetics, Maternal and Child Health, University of Genoa, Genoa 16132, Italy; Department of Neuroscience, Rehabilitation, Ophthalmology, Genetics, Maternal and Child Health, University of Genoa, Genoa 16132, Italy; Department of Neuroscience, Rehabilitation, Ophthalmology, Genetics, Maternal and Child Health, University of Genoa, Genoa 16132, Italy; Department of Neuroscience, Rehabilitation, Ophthalmology, Genetics, Maternal and Child Health, University of Genoa, Genoa 16132, Italy; Department of Neuroscience, Rehabilitation, Ophthalmology, Genetics, Maternal and Child Health, University of Genoa, Genoa 16132, Italy; Obstetrics and Gynecology Unit, IRCCS Istituto Giannina Gaslini, Genoa 16147, Italy; Department of Neuroscience, Rehabilitation, Ophthalmology, Genetics, Maternal and Child Health, University of Genoa, Genoa 16132, Italy; Pediatric Endocrinology Unit, Department of Pediatrics, IRCCS Istituto Giannina Gaslini, Genoa 16147, Italy; Pediatric Endocrinology Unit, Department of Pediatrics, IRCCS Istituto Giannina Gaslini, Genoa 16147, Italy; Pediatric Endocrinology Unit, Department of Pediatrics, IRCCS Istituto Giannina Gaslini, Genoa 16147, Italy; Pediatric Endocrinology Unit, Department of Pediatrics, IRCCS Istituto Giannina Gaslini, Genoa 16147, Italy; Department of Neuroscience, Rehabilitation, Ophthalmology, Genetics, Maternal and Child Health, University of Genoa, Genoa 16132, Italy; Department of Neuroscience, Rehabilitation, Ophthalmology, Genetics, Maternal and Child Health, University of Genoa, Genoa 16132, Italy; Pediatric Endocrinology Unit, Department of Pediatrics, IRCCS Istituto Giannina Gaslini, Genoa 16147, Italy; Pediatric Endocrinology Unit, Department of Pediatrics, IRCCS Istituto Giannina Gaslini, Genoa 16147, Italy; Department of Neuroscience, Rehabilitation, Ophthalmology, Genetics, Maternal and Child Health, University of Genoa, Genoa 16132, Italy; Pediatric Endocrinology Unit, Department of Pediatrics, IRCCS Istituto Giannina Gaslini, Genoa 16147, Italy; Department of Neuroscience, Rehabilitation, Ophthalmology, Genetics, Maternal and Child Health, University of Genoa, Genoa 16132, Italy

**Keywords:** precocious puberty, body composition, adiposity, dual-energy X-ray absorptiometry

## Abstract

**Objective:**

Distinguishing central precocious or early puberty (CPP/EP) from nonprogressive premature breast development (npPBD) is challenging, particularly in the context of increasing childhood adiposity, which may affect growth and skeletal maturation independently of hypothalamic-pituitary-ovarian (HPO) axis activation.

**Methods:**

We retrospectively evaluated 100 girls (69 CPP/EP, 31 npPBD) and 16 age-matched prepubertal girls with obesity as controls. Assessments included auxology, bone age, hormonal and metabolic profiles, pelvic ultrasound, DXA-derived body composition, and lifestyle factors.

**Results:**

Anthropometric parameters and bone age were similar between CPP/EP and npPBD, whereas LH, FSH, estradiol, and IGF-1 SDS (all *P* < .001) remained the main discriminators between groups. Overall body composition was comparable, except for a higher android-to-gynoid fat ratio in npPBD (*P* = .03). In both diagnostic groups, overweight/obesity was associated with greater height relative to target height (*P* = .004 in CPP/EP; *P* = .03 in npPBD) and higher IGF-1 SDS (*P* < .001 and *P* = .02, respectively) compared with controls. Among normal-weight girls, those with CPP/EP showed greater height relative to target height (*P* = .009) and more advanced bone age (*P* = .02) than those with npPBD, whereas these differences were not observed in girls with overweight/obesity.

**Conclusion:**

Excess adiposity reduces the diagnostic specificity of auxological and skeletal maturation parameters in girls with early breast development. Biochemical assessment remains central, with IGF-1 SDS providing additional supportive information.

Distinguishing central precocious puberty (CPP) from nonprogressive premature breast development is clinically challenging, particularly in the context of rising childhood obesity and a secular trend toward earlier pubertal onset [1, 2]. Recent evidence indicates that lifestyle-related increases in adiposity have contributed to a surge in referrals for suspected precocious puberty, a pattern that became especially pronounced during the COVID-19 pandemic [3, 4]. Most studies report that 85-99% of CPP cases in girls are idiopathic [5, 6], with organic lesions found in 0-10%, mostly before age 6 [6-9], and a small subset linked to genetic mutations, particularly in familial or early-onset cases. Inactivating mutations in MKRN3 and DLK1 are the most common monogenic causes identified to date [10-13]. This has redirected attention to modifiable factors such as adiposity, low physical activity, poor sleep, and screen use as potential drivers of the observed increase in idiopathic CPP. CPP is characterized by premature activation of the HPO axis with progressive pubertal development, whereas breast development without sustained hormonal activation or pubertal progression (npPBD) is generally benign. In the literature, this condition is often described within the spectrum of non or slowly progressive precocious puberty, as opposed to rapidly progressive forms requiring intervention [14-16]. Several studies have shown that girls with non or slowly progressive puberty represent a benign variant, typically showing normal pubertal progression and favorable outcomes, including normal age at menarche and adult height [14, 17, 18]. Accurate differentiation is therefore essential, as CPP may warrant intervention and longitudinal monitoring, while npPBD is usually managed conservatively. Excess adiposity has been consistently associated with earlier pubertal onset in girls, suggesting that pubertal initiation is closely linked to energy availability and metabolic signaling. Adipose-derived factors such as leptin have been implicated in activating the HPO axis [19], and emerging evidence points to additional lipid-mediated pathways, including ceramide signaling, that may influence pubertal timing [20]. Importantly, both obesity and early puberty have been associated with adverse long-term metabolic outcomes [21, 22], underscoring the need for careful clinical evaluation of pubertal signs in overweight and obese children.

Body mass index (BMI) is routinely used to assess obesity in clinical practice; however, it does not capture differences in fat distribution or lean mass that may influence growth patterns and pubertal staging [23]. In contrast, dual-energy X-ray absorptiometry (DXA) provides a precise assessment of total and regional body composition [24], which may offer complementary information to contextualize adiposity-related growth patterns in girls evaluated for early pubertal signs.

In our previous DXA-based study, we showed that girls with central precocious or early puberty and premature thelarche may exhibit partially distinct body composition and bone phenotypes [25]; however, the independent contribution of adiposity to these differences remains incompletely defined.

In addition, lifestyle factors such as physical activity and dietary behaviors, which may modulate both adiposity and pubertal timing, have not been systematically examined in relation to CPP and npPBD.

Therefore, the primary aim of this study was to compare clinical, hormonal, auxological, and DXA-derived body composition characteristics between girls with CPP/EP and those with npPBD, in order to determine whether adiposity and HPO-axis activation are associated with distinct phenotypic patterns that may support clinical differentiation. As a secondary objective, we explored lifestyle-related factors to assess their potential contribution to these phenotypes.

## Materials and methods

**Figure 1:**
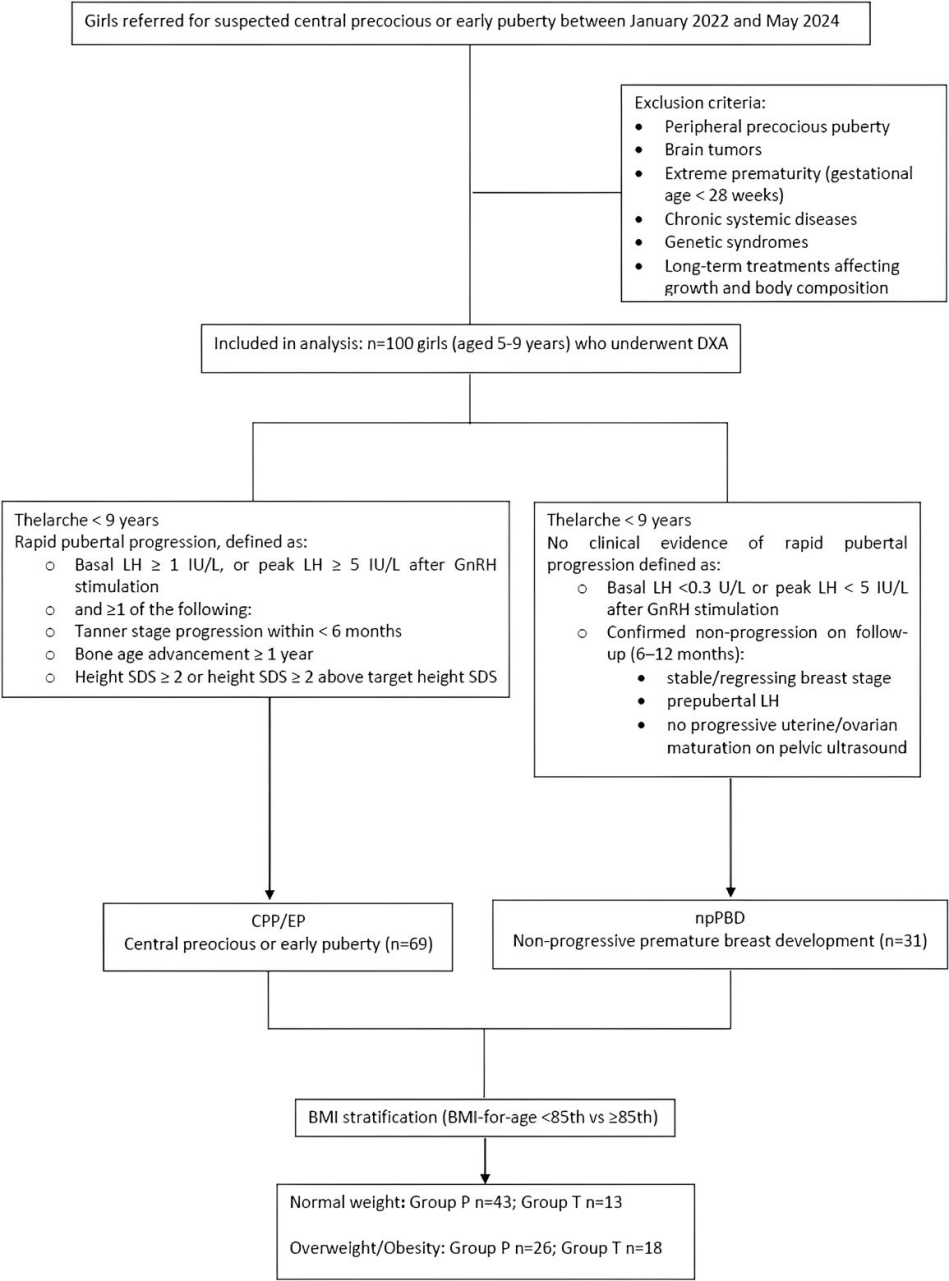
Flowchart illustrating the selection of 100 girls from all those referred for suspected early pubertal development who underwent DXA between January 2022 and May 2024. After applying exclusion criteria, 69 were classified with central precocious/early puberty (CPP/EP) and 31 with non-progressive premature breast development (npPBD) after a 6–12-month observation period.

This retrospective observational single-center study was conducted at the Pediatric Endocrine Unit of IRCCS Istituto Giannina Gaslini (University of Genoa, Italy). We reviewed the medical records of 100 girls aged 5-9 years who were referred for suspected CPP or early pubertal development and who underwent DXA as part of their clinical evaluation between January 2022 and May 2024 (Fig. 1). DXA assessments were performed at diagnosis or within 3 months of the initial endocrine evaluation.

Sixty-nine girls met the diagnostic criteria for central precocious puberty (CPP) or early puberty (EP) and were classified as CPP/EP. CPP was defined as the onset of breast development at Tanner stage ≥2 before 8 years of age, whereas EP was defined as pubertal onset between 8 and 9 years of age [26]. The diagnosis was established based on clinical signs and biochemical evidence of hypothalamic–pituitary–ovarian (HPO) axis activation, defined as basal serum luteinizing hormone (LH) > 1 U/L and/or peak LH >5 U/L after a gonadotropin-releasing hormone (GnRH) stimulation test. LH was measured using a chemiluminescent immunoassay with functional sensitivity in the prepubertal range. Biochemical activation had to be associated with at least one of the following auxiliary criteria: Tanner stage progression within <6 months, bone age advancement ≥1 year relative to chronological age and/or height (H), or delta height minus target height (ΔH–TH) ≥ +2 standard deviation scores (SDS) [27, 28]. These combined features are consistent with the definition of rapidly progressive central precocious or early puberty [28]. Girls with CPP and EP were analyzed together because both conditions reflect premature central activation of the HPO axis and share comparable clinical implications.

Thirty-one girls were classified as nonprogressive premature breast development (npPBD) after a 6-12-month observation period. All had onset of breast development (breast Tanner stage ≥2) before 9 years of age and a prepubertal gonadotropin profile at initial evaluation (basal LH <0.3 U/L and/or GnRH-stimulated peak LH <5 U/L, when performed). Nonprogression was confirmed by a stable or regressing breast Tanner stage, persistently prepubertal gonadotropins (basal LH <0.3 U/L), and no evidence of progressive uterine or ovarian maturation on pelvic ultrasound. Overall diagnostic classification was based on an integrated clinical, auxological, radiological, and biochemical assessment. According to routine practice at our center, the GnRH stimulation test was performed when clinically indicated, including in the presence of progressive pubertal signs, accelerated growth velocity, advanced bone age, and/or basal gonadotropin levels suggestive of central activation. In girls with a clearly pubertal basal hormonal profile (basal LH >1 U/L) in combination with evidence of rapid progression (breast Tanner stage 3-4, height SDS ≥ 2 and/or ΔH–TH ≥ +2 SDS, and bone age advanced by ≥1 year relative to chronological age), GnRH stimulation testing was considered unnecessary. Conversely, in girls with premature breast development whose basal LH levels were not suggestive of central activation and who did not show clinical evidence of progressive puberty, a conservative strategy with longitudinal follow-up was adopted, and GnRH stimulation testing was not routinely required. The 2 girls in this group who did not undergo GnRH stimulation testing had prepubertal basal gonadotropin levels and showed no evidence of pubertal progression during follow-up. Auxological growth acceleration and bone age advancement were not used as exclusion criteria for npPBD, as these may occur in the context of excess adiposity even in the absence of demonstrable central HPO-axis activation. Exclusion criteria included peripheral precocious puberty, central nervous system tumors, extreme prematurity (<28 weeks' gestation), and medical conditions or long-term medications known to independently affect growth and body composition (eg, major endocrine disorders, chronic systemic diseases, or genetic syndromes).

We included a control group of 16 prepubertal girls with essential obesity, all younger than 9 years at clinical evaluation and without breast development (breast Tanner stage: 1). Basal LH levels were consistent with the absence of HPO axis activation; therefore, neither a GnRH stimulation test nor a pelvic ultrasound was performed. All girls underwent DXA between 2009 and 2025 during a clinical evaluation for excess weight. At the time of evaluation, none were receiving pharmacological treatment for obesity, and all were managed exclusively with dietary and behavioral interventions. None met the predefined exclusion criteria.

The study was approved by the local Ethics Committee (Protocol No. 13777/21; CER, Genoa, Italy). Written informed consent was obtained from parents or legal guardians in accordance with the Declaration of Helsinki.

## Data collection

### Historical and lifestyle data

Historical data included age at thelarche, maternal age at menarche, gestational age at birth, birth weight, and ethnicity (classified as European or non-European). Lifestyle-related information was collected through structured parental interviews and included sleep schedules and electronic device use. Parents were asked to report bedtime and wake time on weekdays and weekends, average daily screen time, type of electronic devices used, and whether the child owned a personal electronic device.

### Questionnaires

Adherence to the Mediterranean diet, physical activity levels, and sleep behavior were assessed using validated Italian versions of the KIDMED questionnaire [29], the Physical Activity Questionnaire for Older Children (PAQ-C) [30], and the Sleep Disturbance Scale for Children (SDSC) [31], respectively. Questionnaires were completed during the clinical visit with parental assistance when appropriate.

The KIDMED questionnaire evaluates adherence to the Mediterranean diet and yields a total score ranging from 0 to 12, with scores ≤3 indicating poor adherence, 4-7 moderate adherence, and ≥8 optimal adherence. Following the methodology described by Altavilla et al [32], the item “Takes a fruit or fruit juice every day” was modified to “Takes a fruit every day,” with homemade orange juice accepted as an alternative; scoring and cutoff values were unchanged.

The PAQ-C is a 7-day recall questionnaire designed to assess general physical activity levels in children aged 8-14 years [30]. It consists of 9 items rated on a 5-point scale, resulting in a composite score ranging from 1 (low physical activity) to 5 (high physical activity). The questionnaire was administered to age-eligible participants, with parental assistance provided when needed.

Sleep behavior and disturbances were evaluated using the SDSC, a 27-item Likert-type scale developed to assess sleep disorders in children aged 6-15 years [31]. Parents rated the frequency of sleep-related behaviors on a 5-point scale ranging from “never” to “always.” Higher total scores indicate greater sleep disturbance, and a cutoff score of 39 was used to identify clinically relevant sleep problems, as recommended by the original validation study.

### Clinical data

Pubertal development was assessed by breast inspection and palpation according to Tanner staging criteria [33]. Standing height was measured to the nearest 0.1 cm using a calibrated Harpenden stadiometer, and body weight was measured to the nearest 0.1 kg using an electronic scale, with participants barefoot and wearing light clothing.

Target height (TH) for girls was calculated as (father's height + mother's height − 13 cm)/2 [34] and expressed as SDS based on Tanner–Whitehouse growth references [35]. Height relative to genetic potential was expressed as the difference between measured height SDS and target height SDS (H–TH SDS).

BMI was calculated as weight (kg)/height (m^2^) and converted to SDS using the LMS method based on World Health Organization (WHO) reference charts [36]. For categorical classification, overweight was defined as BMI between the 85th and 97th percentile and obesity as BMI above the 97th percentile, in accordance with the consensus position statement of the Italian Society for Pediatric Endocrinology and Diabetology (ISPED) and the Italian Society of Pediatrics, using the 2007 WHO growth references [37, 38].

### Biochemical data

After an overnight fast, blood samples were collected in the morning for the assessment of metabolic and hormonal parameters.

Triglycerides, total cholesterol, HDL cholesterol, LDL cholesterol, and glycated hemoglobin (HbA1c) were measured using Roche reagents on a Roche Cobas C501 analyzer. Triglycerides were assessed by an enzymatic colorimetric test; total cholesterol by an enzymatic colorimetric assay traceable to ID/MS; HDL cholesterol by a fourth-generation homogeneous enzymatic colorimetric assay; LDL cholesterol was measured directly by a third-generation homogeneous enzymatic colorimetric assay; and HbA1c by TINIA (turbidimetric inhibition immunoassay) standardized according to IFCC. Basal insulin concentrations were determined on a Roche Cobas e801 analyzer using Roche Elecsys reagents by electrochemiluminescence immunoassay (ECLIA).

Basal follicle-stimulating hormone (FSH), luteinizing hormone (LH), and estradiol (E2) levels were measured after venous blood sampling performed between 08:00 and 09:00 Am using an electrochemiluminescence immunoassay (ECLIA) on a Roche Elecsys 2010 analyzer (Roche Diagnostics).

The GnRH stimulation test was performed by intravenous administration of synthetic GnRH (Lutrelef™, Ferring) at a dose of 100 µg/m^2^ (maximum dose 100 µg). Blood samples for LH and FSH determination were obtained at baseline (0 minutes) and at 15, 30, and 60 minutes after GnRH administration.

All serum IGF-1 samples were measured using an electrochemiluminescence immunoassay (ECLIA) (Roche Diagnostics GmbH, Mannheim, Germany; RRID AB_2883975). IGF-1 values were converted to standard deviation scores (SDS) using age- and sex-specific reference data [39, 40].

### Radiological data

Bone age (BA) was assessed from left hand–wrist radiographs using BoneXpert™ based on Greulich [41, 42], and independently verified (D.F.). The difference between bone age and chronological age (ΔBA–A) was calculated accordingly.

Pelvic transabdominal ultrasound was performed with a 2-7 MHz curvilinear probe by an experienced gynecologist (V.T.) to measure uterine dimensions and ovarian size, with ovarian volume calculated using the ellipsoid formula (length × transverse diameter × fundal anteroposterior diameter × 0.5233).

### DXA-derived assessment of body composition parameters

All girls underwent DXA examination with a Lunar iDXA (software version 18; GE Medical Systems Lunar). The DXA machine's calibration was checked daily with a GE Lunar block calibration phantom; the results were within the acceptable range of variations.

Two trained operators (N.D.I. and A.A.) performed and quality-checked all DXA scans, correcting region-of-interest (ROI) positioning when needed. ROIs (total body, arms, legs, trunk, android, and gynoid) were automatically defined by enCORE software. Assessed parameters included total fat mass (kg and %), fat-free mass (kg), truncal fat mass (kg and %), and upper- and lower-limb lean mass (kg). The android and gynoid regions were defined automatically using standardized anatomical landmarks, and the android-to-gynoid (A/G) fat ratio was calculated from fat mass within these ROIs [43]. From these measurements, the following body composition indices were calculated: appendicular lean mass index (ALMI = [upper + lower limb lean mass]/height^2^, kg/m^2^), fat mass index (FMI = FM/height^2^, kg/m^2^), and fat-free mass index (FFMI = FFM/height^2^, kg/m^2^) [44]. Fat-free mass (FFM) refers to the total mass excluding all fat tissue. It is calculated by subtracting fat mass from total body weight (FFM = Total Body Weight−Fat Mass).

## Statistical analysis

The distribution of continuous variables was assessed using the Kolmogorov–Smirnov test. As most variables were not normally distributed and subgroup sizes were relatively small and unbalanced, nonparametric tests (Mann–Whitney *U* test) were used for between-group comparisons. Categorical variables were expressed as absolute numbers and percentages and compared using Pearson's χ^2^ test or Fisher's exact test, as appropriate.

Exploratory analyses were additionally stratified by weight status (normal weight vs overweight/obesity) within and across diagnostic groups.

All statistical analyses were performed using the Statistical Package for the Social Sciences (SPSS) for Windows, version 29 (IBM Corp., Armonk, NY, USA). A two-tailed *P*-value <.05 was considered statistically significant.

## Results

### Clinical characteristics

Breast development occurred before 8 years of age in 86.0% of the overall cohort, including 84.1% of girls with central precocious puberty and 90.3% of those with nonprogressive premature breast development, whereas pubertal onset between 8 and 9 years was observed in 14.0% of participants (15.9% and 9.7%, respectively).

Overall, 44.0% of girls were classified as overweight or obese, including 20.0% with obesity. Excess weight (overweight/obesity) was more common in girls with nonprogressive premature breast development than in those with central precocious/early puberty (58.1% vs 37.7%), although this difference did not reach statistical significance (*P* = .081). While the prevalence of overweight was similar between groups (25.8% in npPBD vs 23.2% in CPP/EP; *P* = .803), obesity was more frequent in npPBD than in CPP/EP (32.3% vs 14.5%), without reaching statistical significance (*P* = .058).

Non-European origin was reported in 24.0% of the study population, with a slightly higher proportion among girls with central precocious/early puberty than among those with nonprogressive premature breast development (26.9% vs 19.3%), although this difference was not statistically significant; 10.0% of the overall cohort was of Honduran descent. In the control group, 18.8% of the girls were of non-European origin.

A GnRH stimulation test was performed in 78.3% of girls with CPP/EP and was omitted in those with clearly pubertal baseline findings (basal LH >1 U/L) accompanied by advanced clinical/auxological features, including breast Tanner stage 3-4 and/or marked growth or skeletal advancement (height SDS or height relative to target height ≥+2 SDS and/or bone age advancement ≥1 year). In girls with npPBD, GnRH testing was performed in 93.5% of cases, confirming the absence of biochemical HPO-axis activation (peak LH <5 U/L). The 2 girls in this group who did not undergo GnRH stimulation testing had prepubertal basal gonadotropin levels and showed no evidence of rapid pubertal progression during follow-up.

In npPBD, follow-up over 6-12 months showed regression of breast Tanner stage in 6/31 girls (19.4%), whereas breast development remained stable in 25/31 (80.6%). Among those with stable breast development, 7 girls exhibited increased growth velocity, ranging from 1.6 to 2.1 SDS, whereas the others showed lower growth velocity. Across the entire group, basal LH remained <0.3 U/L on repeated measurements, and pelvic ultrasonography did not show progressive uterine or ovarian maturation, supporting the absence of sustained central activation during the observation window.

### Auxological, hormonal, and body composition comparison between girls with CPP/EP and those with npPBD

We first compared CPP/EP and npPBD as whole groups, and subsequently performed analyses stratified by weight status and comparisons with prepubertal controls with obesity.

The 2 groups did not differ significantly in age at thelarche, age at evaluation, height, weight, or BMI, as shown in Table 1. Bone age advancement was comparable between groups, although a nonsignificant tendency toward greater advancement was observed in CPP/EP. Maternal age at menarche was significantly lower in CPP/EP compared with npPBD (*P* = .01).

**Table 1:** Comparison of clinical, biochemical, ultrasound, and densitometric characteristics between girls with CPP/EP and those with npPBD

Parameter	CPP/EP (*n* = 69)	npPBD (*n* = 31)	*P*-value
**Clinical and biochemical characteristics**	***Mean ± SD***		
Age at visit (yrs)	7.98 ± 0.83	7.68 ± 1.11	.23
Age at thelarche (yrs)	6.88 ± 1.29	6.97 ± 1.14	.83
Maternal age at menarche (yrs)	11.37 ± 1.71	12.43 ± 2.07	.**01**
Gestational age at birth (weeks)	38.96 ± 2.34	39.52 ± 1.57	.57
Birth weight SDS	0.03 ± 1.07	−0.14 ± 1.07	.63
Height SDS	1.47 ± 1.06	1.42 ± 1.08	.64
Weight (kg)	31.63 ± 6.48	32.54 ± 8.13	.70
BMI SDS	0.86 ± 0.97	1.21 ± 1.12	.09
Target height SDS	−0.34 ± 0.74	−0.07 ± 0.79	.12
Delta H-TH SDS	1.85 ± 1.09	1.50 ± 1.04	.11
Age at bone age evaluation (A) (yrs)	7.81 ± 0.91	7.49 ± 1.13	.23
BA (yrs)	9.50 ± 1.30	8.90 ± 1.47	.07
Delta BA—A(yrs)	1.52 ± 1.03	1.22 ± 0.91	.44
Glycated hemoglobin	5.07 ± 0.24	5.03 ± 0.23	.56
Insulin (uU/mL)	11.20 ± 8.89	13.42 ± 9.65	.36
Total cholesterol (mg/dL)	149.92 ± 20.58	148.67 ± 23.68	.50
LDL cholesterol (mg/dL)	83.52 ± 17.77	82.78 ± 17.61	.76
HDL cholesterol (mg/dL)	60.71 ± 12.93	59.52 ± 17.66	.48
Triglycerides (mg/dL)	61.45 ± 24.27	66.53 ± 29.82	.62
**Hormonal and ultrasound data**	***Mean ± SD***		
Baseline LH (U/L)	1.42 ± 1.63	0.04 ± 0.12	**<**.**001**
Baseline FSH (U/L)	4.52 ± 2.31	1.91 ± 1.09	**<**.**001**
Peak LH (U/L)	12.82 ± 13.08	2.76 ± 1.15	**<**.**001**
Peak FSH (U/L)	13.71 ± 5.32	10.33 ± 3.23	.**004**
Basal LH-FSH ratio	0.28 ± 0.27	0.02 ± 0.06	**<**.**001**
Peak LH-FSH ratio	1.04 ± 0.80	0.27 ± 0.12	**<**.**001**
E2 (pg/mL)	24.98 ± 23.05	5.32 ± 6.71	**<**.**001**
IGF1 SDS	2.89 ± 1.35	1.78 ± 1.15	**<**.**001**
IGF-1 SDS adjusted for BA	1.62 ± 1.01	1.04 ± 0.98	.**01**
Uterus length (mm)	35.19 ± 9.34	32.17 ± 6.76	.28
Uterus AP diameter (mm)	10.59 ± 4.55	8.98 ± 5.96	.**02**
Right ovary volume (cm^3^)	1.54 ± 1.04	1.92 ± 1.57	.35
Left ovary volume (cm^3^)	1.56 ± 1.65	1.88 ± 1.94	.51
**DXA-derived data**	***Mean ± SD***		
Android-gynoid fat ratio	0.29 ± 0.09	0.34 ± 0.11	.**03**
Total fat mass (kg)	9.75 ± 3.90	10.91 ± 4.57	.29
Total fat-free mass (kg)	18.67 ± 3.09	18.10 ± 3.75	.21
Body fat %	34.23 ± 6.89	37.21 ± 7.58	.07
Truncal fat %	29.20 ± 8.64	33.33 ± 9.66	.06
Android fat %	27.21 ± 10.50	31.90 ± 11.81	.06
Gynoid fat %	37.79 ± 6.13	39.88 ± 6.87	.11
FMI (kg/m^2^)	5.42 ± 1.96	6.22 ± 2.18	.08
Upper limbs lean mass (kg)	1.88 ± 0.36	1.87 ± 0.43	.57
Lower limbs lean mass (kg)	6.78 ± 1.28	6.57 ± 1.59	.50
ALMI (kg/m^2^)	4.78 ± 0.54	4.87 ± 0.65	.67
FFMI (kg/m^2^)	10.91 ± 0.94	10.91 ± 1.13	.77

Bold values indicate statistically significant *P*-values (*P* < .05).

Abbreviations: ALMI, appendicular lean mass index; AP, anteroposterior; BA, bone age; delta BA-A, delta bone age-chronological age; E2, estradiol; FFMI, fat-free mass index; FMI, fat mass index; H, height; IGF1, Insulin-Like Growth Factor 1; TH, target height.

**Figure 2:**
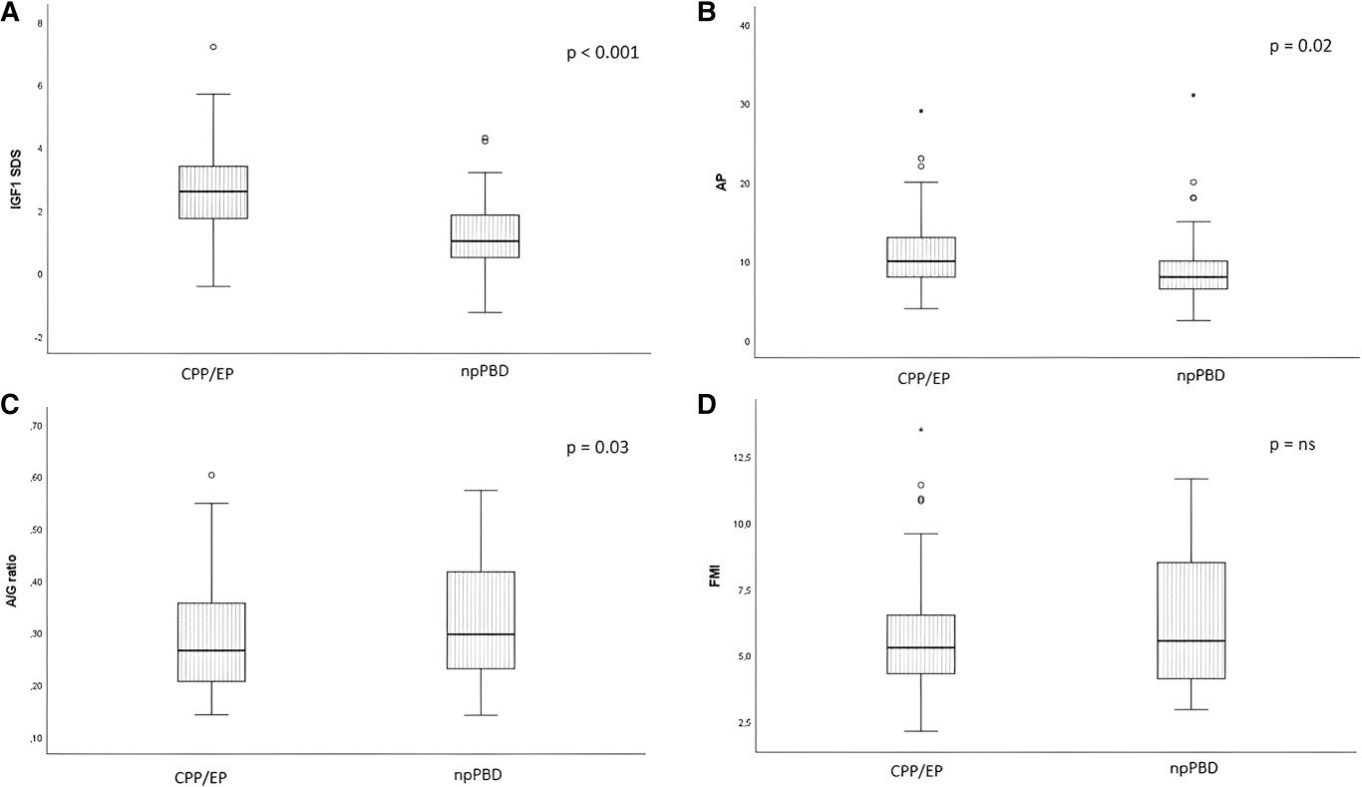
Hormonal, pelvic ultrasound, and body composition parameters in girls with central precocious/early puberty (CPP/EP) and non-progressive premature breast development (npPBD). (A) IGF-1 SDS, (B) uterine anteroposterior (AP) diameter, (C) android-to-gynoid fat ratio (A/G ratio), and (D) fat mass index (FMI). Boxplots show median, interquartile range, and outliers.

With respect to biochemical parameters, no significant differences were observed between groups, except for IGF-1. IGF-1 SDS was significantly higher in girls with CPP/EP than in those with npPBD (mean 2.89 vs 1.78, approximately 62% higher; *P* < .001), and this difference remained significant after adjustment for bone age (*P* = .01) (Fig. 2A).

Pelvic ultrasonography revealed a larger uterine anteroposterior diameter in girls with CPP/EP than in those with npPBD (*P* = .02) (Fig. 2B), whereas other uterine and ovarian measurements did not differ between groups.

Overall body composition assessed by DXA was comparable between groups. However, the android-to-gynoid (A/G) fat ratio was significantly higher in npPBD (0.336 vs 0.287, approximately 17% higher; *P* = .03) (Fig. 2C). Girls with npPBD also showed nonsignificant trends toward higher total, trunk, and android fat percentages, as well as FMI (Fig. 2D). No significant differences between-group were observed in lean mass, FFMI, or ALMI.

### Auxological, hormonal, and body composition comparison between girls with overweight/obesity and normal-weight girls within CPP/EP and npPBD

**Figure 3:**
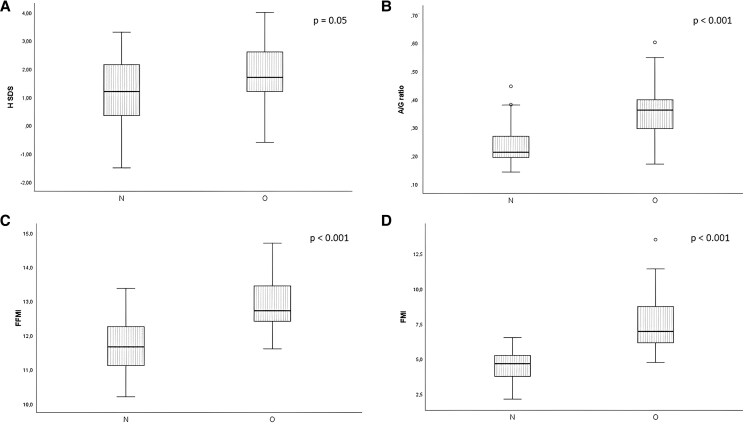
Auxological and body composition parameters in girls with central precocious/early puberty (CPP/EP) according to weight status. Comparison between girls with normal weight (N) and girls with overweight/obesity (O) for (A) height SDS, (B) android-to-gynoid fat ratio (A/G ratio), (C) fat-free mass index (FFMI), and (D) fat mass index (FMI). Boxplots represent median, interquartile range, and outliers.

**Figure 4:**
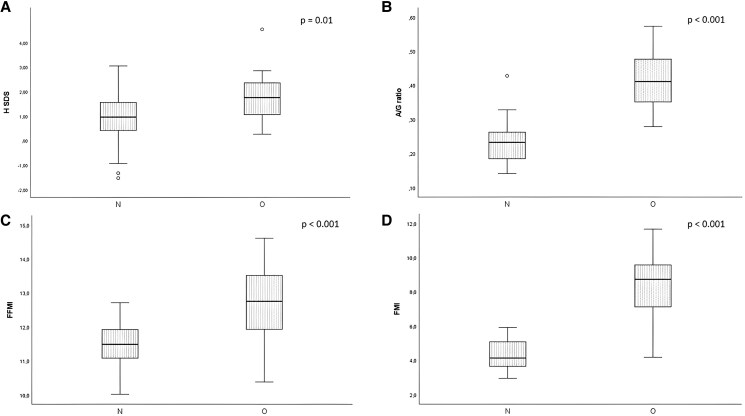
Auxological and body composition parameters in girls with non-progressive premature breast development (npPBD) according to weight status. Comparison between girls with normal weight (N) and girls with overweight/obesity (O) for (A) height SDS, (B) android-to-gynoid fat ratio (A/G ratio), (C) fat-free mass index (FFMI), and (D) fat mass index (FMI). Boxplots represent median, interquartile range, and outliers.

Given the observed tendency toward greater adiposity in npPBD, exploratory subgroup analyses were performed according to weight status (normal weight vs overweight/obesity) within each diagnostic group (Table 2; Figs. 3 and 4).

**Table 2:** Comparison of clinical, biochemical, ultrasound, and densitometric characteristics in girls with CPP/EP and npPBD, stratified by weight category

	CPP/EPP			npPBD		
Parameter	Normal-weight (*n* = 43)	Overweight/obesity (*n* = 26)	*P*-value	Normal-weight (*n* = 13)	Overweight/obesity (*n* = 18)	*P*-value
***Clinical characteristics***			***Mean ± SD***			
Age at visit (yrs)	7.98 ± 0.87	7.97 ± 0.75	.59	7.62 ± 0.95	7.71 ± 1.23	.49
Age at thelarche (yrs)	6.89 ± 1.30	6.86 ± 1.27	1.00	6.82 ± 1.14	7.08 ± 1.16	.65
Height SDS	1.28 ± 0.99	1.76 ± 1.12	.05	0.86 ± 0.84	1.82 ± 1.07	.**01**
Weight (kg)	28.55 ± 4.47	36.74 ± 6.10	**<**.**001**	25.75 ± 4.11	37.450 ± 6.33	**<**.**001**
BMI SDS	0.26 ± 0.57	1.84 ± 0.60	**<**.**001**	0.04 ± 0.59	2.06 ± 0.38	**<**.**001**
Target height SDS	−0.40 ± 0.76	−0.23 ± 0.71	.56	−0.04 ± 0.93	−0.08 ± 0.71	.75
Delta H-TH SDS	1.68 ± 1.07	2.18 ± 1.06	.08	0.81 ± 0.74	1.97 ± 0.96	.**003**
BA (yrs)	9.33 ± 1.15	9.82 ± 1.48	.11	8.26 ± 1.41	9.36 ± 1.36	.**02**
Delta BA—A(yrs)	1.34 ± 0.85	1.82 ± 1.22	.12	0.63 ± 0.95	1.64 ± 0.60	.**002**
**Hormonal and ultrasound data**			***Mean ± SD***			
Baseline LH (U/L)	1.57 ± 1.70	1.21 ± 1.48	.43	0.08 ± 0.16	0.01 ± 0.07	.42
Baseline FSH (U/L)	4.80 ± 2.54	4.03 ± 1.74	.20	2.36 ± 1.44	1.59 ± 0.61	.09
Peak LH (U/L)	13.96 ± 15.74	11.03 ± 7.16	.61	2.92 ± 1.30	2.65 ± 1.06	.47
Peak FSH (U/L)	14.86 ± 5.21	12.21 ± 5.25	.**03**	10.97 ± 4.42	9.88 ± 2.08	.50
Basal LH-FSH ratio	0.29 ± 0.26	0.28 ± 0.28	.71	0.04 ± 0.08	0.01 ± 0.03	.43
Peak LH-FSH ratio	1.00 ± 0.86	1.08 ± 0.73	.39	0.27 ± 0.14	0.26 ± 0.10	.90
E2 (pg/mL)	28.53 ± 24.92	18.44 ± 17.66	.11	7.76 ± 7.72	3.55 ± 5.42	.13
IGF1 SDS	2.45 ± 1.04	3.53 ± 1.46	.**01**	1.47 ± 0.92	2.02 ± 1.28	.34
IGF-1 SDS adjusted for BA	1.49 ± 0.90	1.80 ± 1.13	.**02**	1.02 ± 0.62	1.04 ± 1.19	.67
Uterus length (mm)	35.13 ± 10.40	35.57 ± 7.39	.67	30.58 ± 5.96	37.36 ± 16.42	.17
Uterus AP diameter (mm)	10.66 ± 4.62	10.48 ± 4.53	.77	8.66 ± 4.69	9.23 ± 6.96	.87
Right ovary volume (cm^3^)	1.65 ± 1.18	1.35 ± 0.71	.63	1.98 ± 1.09	1.88 ± 1.90	.48
Left ovary volume (cm^3^)	1.51 ± 1.77	1.60 ± 1.43	.87	2.35 ± 2.57	1.50 ± 1.21	.50
**DXA-derived data**			***Mean ± SD***			
Android-gynoid fat ratio	0.24 ± 0.06	0.33 ± 0.08	**<**.**001**	0.24 ± 0.07	0.41 ± 0.07	**<**.**001**
Total fat mass (kg)	7.72 ± 2.09	13.10 ± 3.92	**<**.**001**	6.56 ± 1.76	14.04 ± 3.14	**<**.**001**
Total fat-free mass (kg)	17.65 ± 2.89	20.41 ± 2.64	**<**.**001**	15.84 ± 1.97	19.74 ± 3.92	.**003**
Body fat %	31.10 ± 5.28	39.41 ± 6.10	**<**.**001**	29.83 ± 4.23	42.54 ± 4.30	**<**.**001**
Truncal fat %	24.99 ± 6.12	36.10 ± 7.56	**<**.**001**	23.61 ± 4.54	40.35 ± 5.07	**<**.**001**
Android fat %	21.65 ± 6.87	36.20 ± 8.92	**<**.**001**	19.88 ± 4.73	40.56 ± 6.37	**<**.**001**
Gynoid fat %	37.79 ± 6.13	39.88 ± 6.87	**<**.**001**	33.71 ± 5.10	44.32 ± 3.86	**<**.**001**
FMI (kg/m^2^)	4.38 ± 0.94	7.15 ± 2.01	**<**.**001**	4.00 ± 0.82	7.83 ± 1.19	**<**.**001**
Upper limbs lean mass (kg)	1.761 ± 0.326	2.07 ± 0.33	**<**.**001**	1.62 ± 0.21	2.05 ± 0.46	.**006**
Lower limbs lean mass (kg)	6.21 ± 1.07	7.47 ± 1.21	**<**.**001**	5.53 ± 0.85	7.32 ± 1.58	**<**.**001**
ALMI (kg/m^2^)	4.53 ± 0.40	5.21 ± 0.49	**<**.**001**	4.38 ± 0.30	5.22 ± 0.61	**<**.**001**
FFMI (kg/m^2^)	10.46 ± 0.78	11.64 ± 0.69	**<**.**001**	10.12 ± 0.46	11.48 ± 1.13	**<**.**001**

Bold values indicate statistically significant *P*-values (*P* < .05).

Abbreviations: ALMI, appendicular lean mass index; AP, anteroposterior; BA, bone age; delta BA-A, delta bone age-chronological age; E2, estradiol; FFMI, fat-free mass index; FMI, fat mass index; H, height; IGF1, Insulin-Like Growth Factor 1; TH, target height.

Among girls with CPP/EP, those with overweight or obesity had higher height SDS (Fig. 3A) and greater height relative to target height (ΔH–TH SDS) than their normal-weight peers, whereas bone age advancement did not differ significantly between weight categories. From a hormonal perspective, girls with overweight/obesity exhibited a lower peak FSH response to GnRH stimulation (*P* = .03) and higher IGF-1 SDS (*P* = .01) compared with normal-weight girls.

Among girls with nonprogressive premature breast development, overweight or obesity was associated with higher height SDS (*P* = .01) (Fig. 4A) and ΔH–TH SDS (*P* = .003), as well as more advanced bone age (*P* = .02) and greater bone age advancement relative to chronological age (ΔBA–CA; *P* = .002). In contrast to girls with CPP/EP, hormonal parameters, including IGF-1 SDS, did not differ significantly between weight categories among girls with nonprogressive premature breast development.

Pelvic ultrasonographic parameters, including uterine and ovarian size, did not differ between girls with overweight/obesity and their normal-weight counterparts in either diagnostic group.

Regarding DXA-derived body composition, girls with overweight or obesity showed significantly higher fat and lean mass than normal-weight girls in both diagnostic groups, android-to-gynoid fat ratio (Figs. 3B and 4B), FFMI (Figs. 3C and 4C), FMI (Figs. 3D and 4D), and ALMI in both CPP/EP and npPBD.

### Auxological, hormonal, and body composition comparison between girls with CPP/EP or npPBD with overweight/obesity and controls with obesity

We performed an additional analysis comparing girls with overweight/obesity in the CPP/EP and npPBD groups with an age-matched control group of prepubertal girls with obesity (Table 3). Controls showed higher weight and BMI SDS than the CPP/EP and npPBD groups (weight: *P* = .002 and *P* = .004, respectively; BMI SDS: *P* < .001 for both comparisons). Height SDS did not differ between groups; however, girls in both CPP/EP and npPBD groups were significantly taller relative to their genetic target height than controls (*P* = .004 and *P* = .03, respectively). Bone age and the difference between bone and chronological age were similar across groups.

**Table 3:** Comparison of clinical, biochemical, and densitometric characteristics among girls with overweight/obesity in the CPP/EP and npPBD groups and girls with obesity in the prepubertal control group

Parameter	CPP/EPP (*n* = 26)	npPBD (*n* = 18)	Controls (*n* = 16)	CPP/EPP vs controls *P*-value	npPBD vs controls *P*-value
***Clinical characteristics***	***Mean ± SD***				
Age at visit (yrs)	7.97 ± 0.75	7.71 ± 1.23	7.93 ± 0.72	.93	.99
Age at thelarche (yrs)	6.86 ± 1.27	7.08 ± 1.16	N.A.	N.A.	N.A.
Height SDS	1.76 ± 1.12	1.82 ± 1.07	1.30 ± 1.08	.23	.30
Weight (kg)	36.74 ± 6.10	37.45 ± 6.33	44.87 ± 8.35	.**002**	.**004**
BMI SDS	1.84 ± 0.60	2.06 ± 0.38	3.07 ± 0.74	**<**.**001**	**<**.**001**
Target height SDS	−0.23 ± 0.71	−0.08 ± 0.71	0.05 ± 0.56	.17	.58
Delta H-TH SDS	2.18 ± 1.06	1.97 ± 0.96	1.16 ± 0.90	.**004**	.**03**
Age at bone age evaluation (A) (yrs)	9.82 ± 1.48	9.36 ± 1.36	8.00 ± 0.72	.34	.49
Bone age (BA) (yrs)	1.82 ± 1.22	1.64 ± 0.60	9.53 ± 1.08	.30	.86
Delta BA—A (yrs)	7.97 ± 0.75	7.71 ± 1.23	1.53 ± 1.07	.54	.31
***Endocrine data***	***Mean ± SD***				
Baseline LH (U/L)	1.21 ± 1.48	0.01 ± 0.07	0.00 ± 0.00	**<**.**001**	.80
Baseline FSH (U/L)	4.03 ± 1.74	1.59 ± 0.61	1.38 ± 0.72	**<**.**001**	.40
Peak LH (U/L)	11.03 ± 7.16	2.65 ± 1.06	N.A.	N.A.	N.A.
Peak FSH (U/L)	12.21 ± 5.25	9.88 ± 2.08	N.A.	N.A.	N.A.
Basal LH-FSH ratio	0.28 ± 0.28	0.01 ± 0.03	0.00 ± 0.00	**<**.**001**	.77
Peak LH-FSH ratio	1.08 ± 0.73	0.26 ± 0.10	N.A.	N.A	N.A
E2 (pg/mL)	18.44 ± 17.66	3.55 ± 5.42	3.06 ± 5.70	.**01**	.80
IGF1 SDS	3.53 ± 1.46	2.02 ± 1.28	0.78 ± 1.04	**<**.**001**	.**02**
IGF-1 SDS adjusted for BA	1.80 ± 1.13	1.04 ± 1.19	−0.05 ± 1.17	**<**.**001**	.**03**
***Densitometric data***	***Mean ± SD***				
Android-gynoid fat ratio	0.33 ± 0.08	0.41 ± 0.07	0.45 ± 0.09	**<**.**001**	.19
Total fat mass (kg)	13.10 ± 3.92	14.04 ± 3.14	20.15 ± 5.15	**<**.**001**	**<**.**001**
Total free fat mass (kg)	20.41 ± 2.64	19.74 ± 3.92	19.52 ± 3.60	.25	.77
Body fat %	39.41 ± 6.10	42.54 ± 4.30	49.0 ± 3.93	**<**.**001**	**<**.**001**
Truncal fat %	36.10 ± 7.56	40.35 ± 5.07	47.0 ± 5.58	**<**.**001**	.**005**
Android fat %	36.20 ± 8.92	40.56 ± 6.37	48.72 ± 6.87	**<**.**001**	.**002**
Gynoid fat %	39.88 ± 6.87	44.32 ± 3.86	50.52 ± 3.22	**<**.**001**	**<**.**001**
FMI	7.15 ± 2.01	7.83 ± 1.19	11.59 ± 2.90	**<**.**001**	**<**.**001**
Upper limbs lean mass (kg)	2.07 ± 0.33	2.05 ± 0.46	2.16 ± 0.44	.64	.60
Lower limbs lean mass (kg)	7.47 ± 1.21	7.32 ± 1.58	7.72 ± 1.26	.53	.53
ALMI	5.21 ± 0.49	5.22 ± 0.61	5.65 ± 0.52	.**01**	.08
FFMI	11.64 ± 0.69	11.48 ± 1.13	11.65 ± 1.07	.70	.99

Bold values indicate statistically significant *P*-values (*P* < .05).

Abbreviations: ALMI, appendicular lean mass index; BA, bone age; delta BA-A, delta bone age-chronological age; E2, estradiol; FFMI, fat-free mass index; FMI, fat mass index; H, height; IGF1, Insulin-Like Growth Factor 1; N.A., not available; TH, target height.

Basal LH, FSH, and estradiol levels did not differ between the npPBD and control group. IGF-1 SDS was higher in both the CPP/EP (*P* < .001) and npPBD groups (*P* = .02), and remained significant after adjustment for bone age (*P* < .001 and *P* = .03, respectively).

Regarding body composition, controls exhibited higher total fat mass, greater total, trunk, gynoid, and android fat percentages, and higher FMI than both patient groups (all *P* < .001). Their android-to-gynoid fat ratio was similar to that observed in the npPBD group but significantly higher than in the CPP/EP group (*P* < .001). Controls also showed significantly higher ALMI than girls with CPP/EP (*P* = .01), with a similar trend compared with the npPBD group.

### Auxological, hormonal, and body composition comparison between CPP/EP and npPBD according to weight status

Additional comparisons between diagnostic groups were performed within each weight category (normal weight vs overweight/obesity) (Table 4).

**Table 4:** Comparison of clinical, biochemical, ultrasound, and densitometric characteristics between girls with CPP/EP and those with npPBD, stratified by weight status

	Normal-weight			Overweight/obesity		
Parameter	CPP/EP (*n* = 43)	npPBD (*n* = 13)	*P*-value	CPP/EP (*n* = 26)	npPBD (*n* = 18)	*P*-value
**Clinical characteristics**		***Mean ± SD***				
Age at visit (yrs)	7.98 ± 0.87	7.62 ± 0.95	.20	7.97 ± 0.75	7.71 ± 1.23	.83
Age at thelarche (yrs)	6.89 ± 1.30	6.82 ± 1.14	.88	6.86 ± 1.27	7.08 ± 1.16	.62
Height SDS	1.28 ± 0.99	0.86 ± 0.84	.08	1.76 ± 1.12	1.82 ± 1.07	.83
Weight (kg)	28.55 ± 4.47	25.75 ± 4.11	.**03**	36.74 ± 6.10	37.45 ± 6.33	.58
BMI SDS	0.26 ± 0.57	0.04 ± 0.59	.30	1.84 ± 0.60	2.06 ± 0.38	.09
Target height SDS	−0.40 ± 0.76	−0.04 ± 0.93	.26	−0.23 ± 0.71	−0.08 ± 0.71	.44
Delta H-TH SDS	1.68 ± 1.07	0.81 ± 0.74	.**009**	2.18 ± 1.06	1.97 ± 0.96	.53
BA (yrs)	9.33 ± 1.15	8.26 ± 1.41	.**02**	9.82 ± 1.48	9.36 ± 1.36	.27
Delta BA—A(yrs)	1.34 ± 0.85	0.63 ± 0.95	.**02**	1.82 ± 1.22	1.64 ± 0.60	.66
**Hormonal and ultrasound data**		***Mean ± SD***				
Baseline LH (U/L)	1.57 ± 1.70	0.08 ± 0.16	**<**.**001**	1.21 ± 1.48	0.01 ± 0.07	**<**.**001**
Baseline FSH (U/L)	4.80 ± 2.54	2.36 ± 1.44	**<**.**001**	4.03 ± 1.74	1.59 ± 0.61	**<**.**001**
Peak LH (U/L)	13.96 ± 15.74	2.92 ± 1.30	**<**.**001**	11.03 ± 7.16	2.65 ± 1.06	**<**.**001**
Peak FSH (U/L)	14.86 ± 5.21	10.97 ± 4.42	.**03**	12.21 ± 5.25	9.88 ± 2.08	**.11**
Basal LH-FSH ratio	0.29 ± 0.26	0.04 ± 0.08	.**001**	0.28 ± 0.28	0.01 ± 0.03	**<**.**001**
Peak LH-FSH ratio	1.00 ± 0.86	0.27 ± 0.14	**<**.**001**	1.08 ± 0.73	0.26 ± 0.10	**<**.**001**
E2 (pg/mL)	28.53 ± 24.92	7.76 ± 7.72	.**007**	18.44 ± 17.66	3.55 ± 5.42	.**01**
IGF1 SDS	2.45 ± 1.04	1.47 ± 0.92	.**001**	3.53 ± 1.46	2.02 ± 1.28	**<**.**001**
IGF-1 SDS adjusted for BA	1.49 ± 0.90	1.02 ± 0.62	.12	1.80 ± 1.13	1.04 ± 1.19	.**003**
Uterus length (mm)	35.13 ± 10.40	30.58 ± 5.96	.14	35.57 ± 7.39	37.36 ± 16.42	.91
Uterus AP diameter (mm)	10.66 ± 4.62	8.66 ± 4.69	.08	10.48 ± 4.53	9.23 ± 6.96	.17
Right ovary volume (cm^3^)	1.65 ± 1.18	1.98 ± 1.09	.27	1.35 ± 0.71	1.88 ± 1.90	.83
Left ovary volume (cm^3^)	1.51 ± 1.77	2.35 ± 2.57	.27	1.60 ± 1.43	1.50 ± 1.21	.93
**DXA-derived data**		***Mean ± SD***				
Android-gynoid fat ratio	0.24 ± 0.06	0.24 ± 0.07	.78	0.33 ± 0.08	0.41 ± 0.07	.**03**
Total fat mass (kg)	7.72 ± 2.09	6.56 ± 1.76	.06	13.10 ± 3.92	14.04 ± 3.14	.32
Total fat-free mass (kg)	17.65 ± 2.89	15.84 ± 1.97	.**02**	20.41 ± 2.64	19.74 ± 3.92	.45
Body fat %	31.10 ± 5.28	29.83 ± 4.23	.34	39.41 ± 6.10	42.54 ± 4.30	.06
Truncal fat %	24.99 ± 6.12	23.61 ± 4.54	.42	36.10 ± 7.56	40.35 ± 5.07	.05
Android fat %	21.65 ± 6.87	19.88 ± 4.73	.58	36.20 ± 8.92	40.56 ± 6.37	.06
Gynoid fat %	37.79 ± 6.13	33.71 ± 5.10	.34	39.88 ± 6.87	44.32 ± 3.86	.10
FMI (kg/m^2^)	4.38 ± 0.94	4.00 ± 0.82	.21	7.15 ± 2.01	7.83 ± 1.19	.06
Upper limbs lean mass (kg)	1.76 ± 0.33	1.62 ± 0.21	.09	2.07 ± 0.33	2.05 ± 0.46	.98
Lower limbs lean mass (kg)	6.22 ± 1.07	5.53 ± 0.85	.**03**	7.47 ± 1.21	7.32 ± 1.58	.81
ALMI (kg/m^2^)	4.53 ± 0.40	4.38 ± 0.30	.23	5.21 ± 0.49	5.22 ± 0.61	.77
FFMI (kg/m^2^)	10.46 ± 0.78	10.12 ± 0.46	.30	11.64 ± 0.69	11.48 ± 1.13	.67

Bold values indicate statistically significant *P*-values (*P* < .05).

Abbreviations: ALMI, appendicular lean mass index; AP, anteroposterior; BA, bone age; delta BA-A, delta bone age-chronological age; E2, estradiol; FFMI, fat-free mass index; FMI, fat mass index; H, height; IGF1, Insulin-Like Growth Factor 1; TH, target height.

Among normal-weight girls, those with CPP/EP had higher body weight (*P* = .03) and greater ΔH–TH SDS (*P* = .009) than their peers with npPBD, as well as more advanced bone age (*P* = .02) and higher IGF-1 SDS (*P* = .001); they also showed a nonsignificant trend toward a larger uterine anteroposterior diameter. Regarding DXA-derived body composition, normal-weight girls with CPP/EP showed a nonsignificant trend toward higher total fat mass than those with npPBD, together with significantly greater total lean mass and lower-limb lean mass (*P* = .02 and *P* = .03, respectively).

Among girls with overweight/obesity, height SDS and ΔH–TH SDS did not differ significantly between CPP/EP and npPBD, whereas BMI SDS showed a nonsignificant trend toward higher values in npPBD. Bone age advancement and pelvic ultrasonographic parameters were comparable between groups. In contrast, IGF-1 SDS was significantly higher in CPP/EP than in npPBD (*P* < .001), and this difference remained significant after adjustment for bone age (*P* = .003). Girls with npPBD exhibited a significantly higher android-to-gynoid (A/G) fat ratio than those with CPP/EP (*P* = .03), along with nonsignificant trends toward higher total, truncal, and android fat percentages and a slightly higher FMI.

## Analysis of questionnaires

### KIDMED questionnaire scores

Overall adherence to the Mediterranean diet, assessed using the KIDMED questionnaire, did not differ significantly between groups. In CPP/EP, 21.7% of girls showed low adherence, 59.4% moderate adherence, and 18.9% high adherence, compared with 19.4%, 61.3%, and 19.4%, respectively, in npPBD. No significant differences in KIDMED score distribution were observed according to weight status within either group.

At the item level, daily fruit/fruit juice consumption was reported more frequently in npPBD than in CPP/EP (90.3% vs 63.8%, *P* = .01). In contrast, no significant differences were observed between groups with respect to vegetable intake, fish consumption, frequency of fast-food consumption, or legume intake.

### PAQ-C questionnaire score

A total of 80 PAQ-C questionnaires assessing physical activity levels were completed, including 57 by girls with CPP/EP and 23 by girls with nonprogressive premature breast development. A PAQ-C score <2.5, indicative of low physical activity, was reported in 46.2% of the overall sample, with similar proportions in CPP/EP and nonprogressive premature breast development (49.1% vs 39.1%). Mean PAQ-C scores did not differ significantly between groups (2.5 ± 0.64 vs 2.6 ± 0.43), and no significant differences were observed according to weight status within or across diagnostic groups.

### SDSC questionnaire

Sleep disturbances, assessed using the Sleep Disturbance Scale for Children (SDSC), were identified in 28.0% of the overall cohort, with similar prevalences in CPP/EP (27.5%) and npPBD (29.0%). Complete sleep data were available for 95 girls. On weekdays, 22 girls (23.2%) reported a sleep duration of <9 hours per night, including 25.8% in CPP/EP and 17.2% in npPBD, whereas this was reported by only 5 girls (5.3%) on weekends, all of whom belonged to CPP/EP.

Bedtime after 10:00 Pm was reported by 21 girls on weekdays (24.2% in CPP/EP and 16.7% in npPBD) and by 61 girls on weekends (66.2% in CPP/EP and 60.0% in npPBD). No significant differences were observed between the 2 diagnostic groups in SDSC total scores, weekday or weekend sleep duration, or sleep timing; findings were also comparable across weight categories.

### Screen time

Mean weekday screen time in the overall cohort was 101.4 ± 85.3 minutes/day, with comparable values in girls with CPP/EP and those with npPBD (104.9 ± 92.1 vs 93.2 ± 67.3 minutes/day). Mean weekend screen time was higher (128.9 ± 104.1 minutes/day), again without significant differences between CPP/EP and npPBD (132.5 ± 110.5 vs 120.5 ± 88.6 minutes/day). No significant differences in screen time were observed between diagnostic groups or according to weight status.

Screen exposure exceeding 2 hours/day was reported by 26.3% of girls on weekdays (28.4% in CPP/EP and 21.4% in npPBD) and by 36.5% on weekends (38.8% in CPP/EP and 31.0% in npPBD).

Detailed information on device-use timing was available for 90 girls (65 with CPP/EP and 25 with npPBD). Use of electronic devices within 30 minutes after waking was reported by 31 girls (34.4%), including 35.4% in CPP/EP and 32.0% in npPBD. Use within 30 minutes before bedtime was reported by 46 girls (51.1%), with similar proportions in CPP/EP and npPBD (48.5% vs 58.3%).

Information on device ownership was available for 95 girls (67 with CPP/EP and 28 with npPBD). Overall, 54 (56.8%) owned at least one personal electronic device, including 53.7% in CPP/EP and 64.3% in npPBD. The most owned devices were tablets (40.9%) and televisions (34.4%). Overall, 69.4% of girls reported daily use of at least one electronic device, with televisions being the most frequently used (52%), followed by smartphones (27%) and tablets (17%).

## Discussion

In this study, we compared body composition and lifestyle characteristics in girls with central precocious or early puberty and those with nonprogressive premature breast development, integrating auxological parameters, bone age, pelvic ultrasonography, DXA-derived body composition, and metabolic and hormonal profiles. Stratification by weight status allowed us to explore the relative contributions of HPO-axis activation and excess adiposity to growth patterns and pubertal features. To further clarify the role of adiposity in growth, girls with overweight/obesity in both diagnostic groups were also compared with age-matched prepubertal controls with obesity.

Hormonal markers clearly differentiated CPP/EP from npPBD, whereas anthropometric measures and bone age advancement were broadly similar between groups. Pelvic ultrasonography showed a significantly greater uterine anteroposterior diameter in girls with CPP/EP, supporting its role as a marker of central activation. However, considerable overlap between conditions was observed, as previously reported, likely reflecting the known influence of pubertal maturation, estrogen exposure, and imaging-related variability on uterine dimensions [45, 46].

Excess adiposity was associated with greater stature and height relative to genetic target height in both diagnostic groups, reaching statistical significance only in girls with npPBD, in whom it was also associated with greater bone age advancement.

When the analysis was restricted to girls with overweight/obesity, auxological parameters, including bone age advancement, were comparable between CPP/EP and npPBD. This finding supports the notion that excess adiposity may attenuate differences in skeletal maturation typically associated with HPO-axis activation, thereby reducing the discriminative value of growth and bone age parameters in this setting. Consistently, both groups also showed similar height SDS and bone age compared with age-matched prepubertal controls with obesity, despite HPO-axis activation in CPP/EP. However, both groups showed greater height relative to genetic target height and higher IGF-1 SDS than controls, suggesting that the growth pattern associated with early breast development is not fully explained by excess adiposity alone. By contrast, among normal-weight girls, height relative to target height and bone age advancement were significantly greater in CPP/EP than in npPBD, indicating that the auxological effects of true HPO-axis activation emerge more clearly in the absence of adiposity as a confounding factor.

Overall, these findings support previous evidence that excess adiposity may promote linear growth and skeletal maturation even without central HPO-axis activation [47-49], while indicating that the growth pattern associated with early breast development cannot be attributed to obesity alone.

Despite similar BMI SDS and total fat mass between diagnostic groups, girls with nonprogressive premature breast development showed a higher android-to-gynoid fat ratio, approximately 17% higher overall than in girls with CPP/EP, particularly among those with overweight or obesity, in whom the android-to-gynoid fat ratio was approximately 24% higher in girls with npPBD than in those with CPP/EP, consistent with a more central fat distribution [50, 51]. Among girls with overweight/obesity, the A/G ratio in npPBD was comparable to that observed in age-matched prepubertal controls with obesity, whereas girls with CPP/EP showed significantly lower values.

Taken together, these findings suggest that, in the presence of excess adiposity, body fat distribution in npPBD more closely resembles that of prepubertal girls with obesity, whereas girls with CPP/EP may already show a relatively less central adiposity pattern, possibly reflecting the effects of pubertal maturation on body composition. Importantly, these differences should be interpreted in light of the fact that body fat distribution is influenced by multiple factors beyond gonadal steroids, including insulin sensitivity, adipokine signaling, genetic background, and environmental exposures [52-54].

Beyond fat distribution, differences in overall body composition also emerged. In line with previous studies [55], overweight or obesity was associated with increased fat and lean mass in both diagnostic groups. Among normal-weight girls, those with CPP/EP displayed greater lean mass and a tendency toward higher fat mass, consistent with pubertal increases in both compartments [56] and the anabolic effects of the growth hormone/IGF-1 axis. IGF-1 SDS was consistently higher in girls with CPP/EP than in those with npPBD across weight categories, supporting its value as a complementary marker alongside standard biochemical assessment of HPO-axis activation. Mean IGF-1 SDS values were approximately 67% higher in normal-weight girls (2.45 vs 1.47) and 75% higher in girls with overweight/obesity (3.53 vs 2.02). In girls with CPP/EP and overweight/obesity, higher IGF-1 levels may reflect a synergistic interaction between pubertal activation and adiposity-related metabolic signals [57, 58].

The prevalence of overweight (24%) and obesity (20%) in our cohort exceeded national estimates from the Italian OKkio alla SALUTE surveillance system [59], particularly among girls with nonprogressive premature breast development. Excess adiposity has been consistently linked to earlier breast development. Kaplowitz et al [60] proposed a causal role of adiposity, and Brix et al [61] confirmed that overweight and obesity predict earlier pubertal milestones in sibling-matched analyses. Nevertheless, additional determinants appear to contribute. Aksglaede et al [17] reported that although thelarche advanced over time in Danish girls, menarche timing did not shift to the same extent, suggesting that excess adiposity may anticipate breast development, while progression to full central activation requires further biological or environmental factors.

In our cohort, self-reported lifestyle behaviors, including screen time, physical activity, dietary habits, and sleep, did not differ significantly between girls with CPP/EP and those with nonprogressive premature breast development, nor across weight categories. Average daily screen exposure was approximately 100-130 minutes, and about one-third of participants exceeded 2 hours per day. Although higher adherence to the Mediterranean diet and adequate sleep have been associated with more favorable metabolic outcomes [62, 63], our data indicate that, on the basis of self-reported measures, lifestyle factors alone do not account for the observed differences in HPO-axis activation or DXA-derived body composition. This suggests that additional, intrinsic determinants may contribute to early pubertal phenotypes. Consistently, the earlier maternal age at menarche observed in the CPP/EP group supports a genetic or familial predisposition to earlier pubertal timing [64].

Nonetheless, the absence of lifestyle differences should be interpreted cautiously, as questionnaire-based assessments are subject to recall and reporting bias and may not capture important qualitative aspects of behavior [65-67]. Unmeasured environmental, socioeconomic, and lifestyle-related factors may also play a role, potentially through epigenetic mechanisms [68, 69].

The main strengths of this study include the comprehensive phenotypic characterization of the cohort, combining hormonal profiling, pelvic ultrasonography, anthropometry, and DXA-based assessment of body composition, a gold-standard method [70]. Stratification by weight status helped to better distinguish adiposity-related effects from those associated with HPO-axis activation, providing clinically relevant insights for interpreting auxological and DXA-derived findings. A further strength of this study is the inclusion of an age-matched prepubertal control group with obesity, which helped contextualize the contribution of excess adiposity to growth and skeletal maturation relative to girls with central precocious or early puberty and nonprogressive premature breast development.

Limitations include the retrospective design, relatively small subgroup sizes, and reliance on self-reported lifestyle questionnaires [65-67]. Parental BMI, socioeconomic factors, genetic background, and environmental exposures were not assessed, and the potential influence of non-European ancestry on pubertal timing and body composition could not be specifically evaluated. Although the 8-year cutoff for the larche aligns with ISPED recommendations [71], international data support earlier thresholds in certain populations [17, 72-74], particularly in girls with higher BMI or non-European ancestry, warranting cautious interpretation of our findings. Moreover, diagnostic overlap between nonprogressive premature breast development and slowly progressive central precocious or early puberty cannot be completely excluded, particularly in girls with overweight/obesity and in those showing higher growth velocity despite stable thelarche. However, classification as nonprogressive premature breast development was supported by persistently prepubertal gonadotropin profiles and the absence of clinical and ultrasonographic progression over 6-12 months of follow-up, reducing the likelihood of systematic misclassification. An additional limitation is the relatively small size of the control group, which may limit the strength of comparisons with the study groups. IGF-I was measured at a single time point using a specific immunoassay platform, and both IGF-I concentrations and SDS are assay- and reference-dependent, which may limit comparability across studies. Moreover, IGF-I is influenced by nutritional status and metabolic factors. Although fasting insulin was measured, we lacked comprehensive assessments of insulin sensitivity and hepatic function; therefore, residual confounding cannot be excluded. Finally, although IGF-I SDS was adjusted for bone age, we could not fully account for potential effects of pubertal tempo or subtle differences in pubertal stage at the time of sampling.

In summary, distinguishing central precocious or early puberty from nonprogressive premature breast development remains challenging in the context of increasing childhood obesity. In girls with overweight or obesity, auxological and ultrasonographic findings should be interpreted cautiously and alongside biochemical evidence of HPO-axis activation, as excess adiposity reduces their diagnostic specificity [75]. In equivocal cases, longitudinal follow-up with repeated clinical assessment of pubertal progression and, when indicated, reassessment of the HPO axis (basal and/or stimulated gonadotropins) is warranted to confirm the diagnosis and guide management.

In our cohort, hormonal markers remained the clearest discriminators between CPP/EP and npPBD. However, the consistently higher IGF-1 SDS observed in girls with CPP/EP, particularly among those with overweight/obesity and even after adjustment for bone age, supports its value as a complementary marker in the diagnostic evaluation. In addition, girls with npPBD and excess adiposity exhibited a more adverse fat distribution, suggesting a potentially higher metabolic burden.

Longitudinal studies are needed to clarify how fat distribution, pubertal tempo, and metabolic risk evolve over time and to determine whether nonprogressive premature breast development, although typically benign, may carry long-term metabolic implications when excess adiposity is present. While earlier pubertal timing has been associated with adverse cardiometabolic outcomes later in life, particularly in the context of obesity [22, 76], the long-term metabolic implications of nonprogressive premature breast development remain uncertain.

## Data Availability

Due to patient confidentiality, the study data are not publicly accessible. They are available from the corresponding author upon reasonable request.
